# Broad Spectrum Antiangiogenic Treatment for Ocular Neovascular Diseases

**DOI:** 10.1371/journal.pone.0012515

**Published:** 2010-09-01

**Authors:** Ofra Benny, Kei Nakai, Takeru Yoshimura, Lauren Bazinet, James D. Akula, Shintaro Nakao, Ali Hafezi-Moghadam, Dipak Panigrahy, Pouya Pakneshan, Robert J. D'Amato

**Affiliations:** 1 Vascular Biology Program and Department of Surgery, Children's Hospital Boston, Harvard Medical School, Boston, Massachusetts, United States of America; 2 Department of Ophthalmology, Children′s Hospital Boston, Harvard Medical School, Boston, Massachusetts, United States of America; 3 Department of Ophthalmology, Massachusetts Eye & Ear Infirmary, Harvard Medical School, Boston, Massachusetts, United States of America; Ohio State University, United States of America

## Abstract

Pathological neovascularization is a hallmark of late stage neovascular (wet) age-related macular degeneration (AMD) and the leading cause of blindness in people over the age of 50 in the western world. The treatments focus on suppression of choroidal neovascularization (CNV), while current approved therapies are limited to inhibiting vascular endothelial growth factor (VEGF) exclusively. However, this treatment does not address the underlying cause of AMD, and the loss of VEGF's neuroprotective can be a potential side effect. Therapy which targets the key processes in AMD, the pathological neovascularization, vessel leakage and inflammation could bring a major shift in the approach to disease treatment and prevention. In this study we have demonstrated the efficacy of such broad spectrum antiangiogenic therapy on mouse model of AMD.

**Methods and Findings:**

Lodamin, a polymeric formulation of TNP-470, is a potent broad-spectrum antiangiogenic drug. Lodamin significantly reduced key processes involved in AMD progression as demonstrated in mice and rats. Its suppressive effects on angiogenesis, vascular leakage and inflammation were studied in a wide array of assays including; a Matrigel, delayed-type hypersensitivity (DTH), Miles assay, laser-induced CNV and corneal micropocket assay. Lodamin significantly suppressed the secretion of various pro-inflammatory cytokines in the CNV lesion including monocyte chemotactic protein-1 (MCP-1/Ccl2). Importantly, Lodamin was found to regress established CNV lesions, unlike soluble fms-like tyrosine kinase-1 (sFlk-1). The drug was found to be safe in mice and have little toxicity as demonstrated by electroretinography (ERG) assessing retinal and by histology.

**Conclusions:**

Lodamin, a polymer formulation of TNP-470, was identified as a first in its class, broad-spectrum antiangiogenic drug that can be administered orally or locally to treat corneal and retinal neovascularization. Several unique properties make Lodamin especially beneficial for ophthalmic use. Our results support the concept that broad spectrum antiangiogenic drugs are promising agents for AMD treatment and prevention.

## Introduction

Ocular neovascular-related diseases are associated with vision impairment or vision loss in millions worldwide. The growth of abnormal, leaky blood vessels is a prominent component of several debilitating eye diseases including AMD, proliferative diabetic retinopathy (PDR), and retinopathy of prematurity (ROP). Therefore antiangiogenic strategies could be particularly beneficial in preventing and treating the progression of these diseases. Corneal neovascularization is often the result of inflammation, chemical burns, and conditions related to hypoxia[1], [2]. These conditions are currently treated by indirect angiogenesis inhibitors such as steroids and immunosuppressants[1]. Inhibition of retinal nonvascular disease, such as AMD, has been focused on targeting the angiogenic factor VEGF and these therapies are the current gold standard in the treatment of neovascular AMD.

The most prominent class of VEGF blocking therapy is represented by ranibizumab and bevacizumab. These molecules are derived from a humanized anti-VEGF antibody which has the ability to reduce retinal edema in AMD[3]. While clinical trials have shown a beneficial effect in 30–40% of treated patients[3], [4], in many cases these therapies become less effective over time[5]. VEGF blockade can halt angiogenesis and reduce vessel leakage, but does not cause regression of existing vessels [6]. The effect of VEGF traps is short lived and thus repeated intravitreal injections every 4 weeks is required[4]. Moreover, it has been shown that retinal pigment epithelium (RPE)-derived soluble VEGF has a role in maintenance of the choriocapillaris[7] and that VEGF is neuroprotective in the retina Thus chronic treatment with anti-VEGF therapy may induce long term damage to the retina.

In recent years diverse cellular processes have been implicated in the pathenogenesis of retinal diseases such as AMD or PDR. These processes include oxidative stress, vascular permeability, fibrosis, inflammation and impaired function of RPE[8]. Given the multifactorial nature of neovascularization and the contribution of the microenvironment, the search for more effective therapies to block CNV has shifted toward targeting multiple processes rather than a single pathway[9]. In this study we investigated different components involved in the pathology of AMD using distinct assays for angiogenesis, inflammation and vascular permeability. We investigated the progression of CNV in response to a broad spectrum antiangiogenic drug that was found to directly inhibit ocular neovascularization while also inhibiting inflammatory and angiogenic signals. This type of therapy may offer an improvement over current treatments and an alternative where existing therapies fail.

Lodamin, monometoxy poly(etylen)glycol-poly(lactic acid) (mPEG-PLA)-TNP-470, is water soluble and orally available antiangiogenic drug. Lodamin has reduced TNP-470-releated cerebellar side effects and has been shown to have potent anti-cancer activity and inhibit liver metastases in mice. [10] In this study we investigated its efficacy in ophthalmology and studied the drug's effect on key processes in AMD.

Using the CNV model in mice and rats, our findings demonstrated the potency of Lodamin as a broad-spectrum antiangiogenic ophthalmic drug in inhibiting neovascularization, edema and inflammation by targeting numerous molecular pathways in addition to VEGF. Importantly, we found the antiangiogenic activity of Lodamin to induce the regression of established CNV lesions unlike VEGF trap (Flk-1) that solely inhibited CNV growth. Additionally, the broad activity of Lodamin was found to cause no adverse effects in retinal function as demonstrated with ERG. Its safety and unique activity place it as a leading candidate for treating corneal and retinal neovascular diseases.

## Results

### Lodamin inhibits angiogenesis and macrophage recruitment *in vivo*

**Figure 1:**
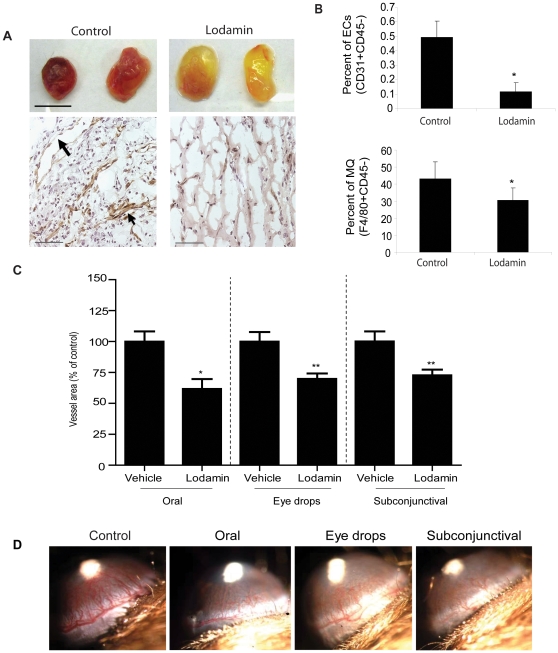
Lodamin inhibits angiogenesis and inflammation in Matrigel plugs and inhibits corneal neovascularization after local or oral treatment. (**A**) Matrigel containing VEGF and bFGF was injected subcutaneously (s.c., n = 5) to determine the effect of Lodamin on angiogenesis and macrophage infiltration. Upper panel shows representative plugs removed from Lodamin treated or untreated mice (bar = 1 cm). Bottom panel shows vessel staining with anti-CD31 antibody (brown) and nuclei staining with Hematoxilin Gill's (blue), bar = 100µm; arrows point to large vessel with open lumans (**B**) Quantification of infiltrating endothelial cell or macrophages in Matrigel plugs. Data are presented as percent of specific cell population out of the total cell population (n = 3–4, p<0.05). (**C**) Antiangiogenic activity of Lodamin following topical or systemic administration was evaluated in the mouse corneal micropocket assay using bFGF-induced neovascularization. Quantification of vessel area (mm^2^) in different corneal assays performed with Lodamin which were administered either orally (30 mg/kg q.d), by eye drops (30 mg/ml, q.d) or by subconjunctival injections (30 mg/ml, q.d). Graphs indicate the significant inhibition of vessel formation after 5 days of treatment. Vessel area was reduced by 38% after subconjunctival injection (*P* = 0.0002), by 30% after eye drops treatment (*P* = 0.003), and by 37% (*P* = 0.04) after oral administration. In each group n = 10. (**D**) Representative images taken from the different groups at day 5; bFGF pellet is detected as a white spot in the center of the cornea, blood vessels growing from limbal periphery are reduced in Lodamin treated group compared to controls. Vessel area in mm^2^ is calculated by the following formula: π×clock hours×vessel length (mm)×0.2 mm.

To investigate Lodamin's effect on angiogenesis and macrophage recruitment, a quantitative mouse Matrigel angiogenesis assay was performed. Vasculature in Matrigel plugs was visually assessed at day 7 post Matrigel injection (Figure. 1 A) and by immunohistochemical staining of blood vessels using anti-CD31 antibodies (Figure 1A). Immunohistology revealed that Lodamin dramatically reduced angiogenesis compared with untreated mice which presented numerous large blood vessels with open lumen structures (n = 5). Infiltrated endothelial cells and macrophages in Matrigel matrices were quantified in a single-cell suspension by fluorescence-activated cell sorting (FACS) (Figure 1B, Figure S1A). After 7 days of oral Lodamin treatment, significant reductions of infiltrating endothelial cells and macrophages were seen in the Lodamin group compared to vehicle treated mice (endothelial cells: 0.11±0.06% versus 0.49±0.11%; macrophages: 30±7% versus 43±9% in Lodamin and control groups, respectively).

### Systemic and local Lodamin treatment inhibit corneal neovascularization

Antiangiogenic activity of Lodamin following topical or systemic administration was evaluated in the mouse corneal micropocket assay using bFGF-induced neovascularization. Mice were treated with Lodamin by oral administration (30 mg/kg/d q.d for 5 days), subconjunctional injection (30 mg/ml q.d for 5 days) or topical eye drops (30 mg/ml q.d for 5 days). These treatments resulted in a 37%, 38% or 30% reduction in corneal vessel area from control respectively. Figure 1C and D shows representative images taken from the designated groups and the quantification of vessel area (Figure 1D).

### Lodamin reduces Delayed-Type Hypersensitivity (DTH) ear-swelling

**Figure 2:**
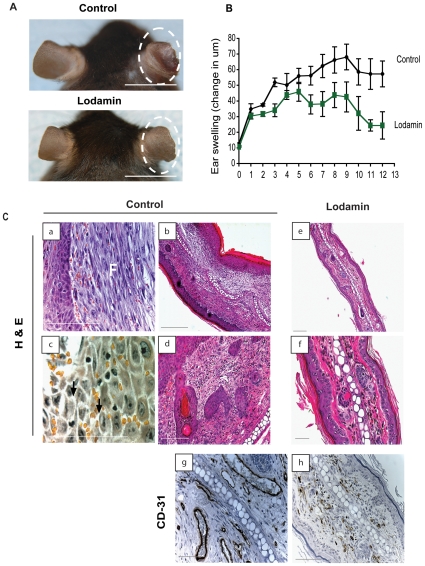
Lodamin inhibits angiogenesis, inflammation and edema in the DTH reaction. (**A**) Lodamin suppressed ear swelling in DTH reactions elicited by oxazolone, photos of representative ears of Lodamin treated or untreated mice are circled. Bar = 1 cm (**B**) Ear swelling is represented as change in thickness (in µm) compared to original ear thickness. Mice treated with Lodamin (white squares) showed a significant reduction in ear thickness compared to control mice (black squares). The differences were statistically significant from day 2 to day 12 post challenge, excluding day 5 (*P*<0.05). Data are presented as an average ±SEM, n = 5. (**C**) Immunohistological analysis of mouse ears post DTH reaction: H&E staining (a–f) and specific staining of endothelial cell marker CD-31 (g–h). In H&H staining, control ears exhibited an excessive inflammation and edema (a–d), including fibroblast proliferation (marked as F) and spongiosis pointed by arrows compared with Lodamin treated mice (e–f). Bar = 50µm.

The multifactorial effect of Lodamin treatment on angiogenesis, inflammation, vascular hyperpermeability and edema was studied in the DTH reaction. C57Bl/6J mice were sensitized with oxazolone and then treated with Lodamin orally (15 mg/kg q.d.) starting from 5 days post initial exposure to oxazolone. As early as day 2 and up to day 12 (with the exceptions of days 4 and 5) a significant reduction of ear erythema and swelling was registered in Lodamin-treated mice compared with vehicle treated mice (Figure 2). Hematoxillin and Eosin (H&E) staining of histological ear sections (Figure 2C,a–f) confirmed markedly reduced swelling and edema in treated mice compared to untreated mice. Unlike the treated mice, control mice demonstrated thick epidermis with extensive edema as illustrated by cell spongiosis, high cellularity and infiltrating monocytes (Figure 2C). A chronic response consisting of neutrophil accumulation and enhanced fibroblast proliferation was also detected in the untreated tissues. In some samples, acute inflammation associated with focal polynuclear cell accumulations was found. Immunostaining of blood vessels with anti-CD31 antibodies revealed significant reduction of angiogenesis by Lodamin (Figure 2C, g–h). By staining with CD45 and F4/80, we also observed reduction of infiltrated leukocytes and macrophages in Lodamin treated mouse ears (Figure S1B).

### Oral or intravitreal treatment of Lodamin reduces CNV progression

**Figure 3:**
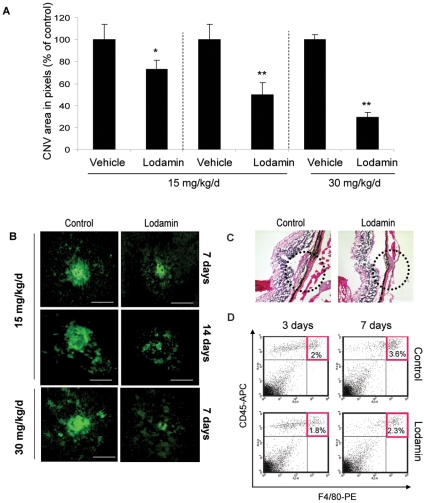
Oral treatment of Lodamin reduces CNV progression. (**A**) CNV lesion size of Lodamin-treated mice compared to controls. A dose of 15 mg/kg/day was administered for 7 or 14 days and a dose of 30 mg/kg/day was administered for 7 days. CNV lesions in choroidal flat mounts were evaluated after staining of blood vessels using isolectin-IB4 conjugated with Alexa Fluor 488. Data are presented as percent of blood vessel area in choroidal flat mounts (pixels) of treatment per controls. Data are expressed as mean ± SEM, (U-test, * *P*<0.05, ** *P*<0.005, n = 10). **(B)** Representative images of CNV lesions stained with a lectin-FITC in flat mount of mouse choroids. Bar = 20 µm. (**C**) H&E stained histological side sections of CNV site in Lodamin treated or untreated mice. Substantial differences in fibrous tissues thickness and choroidal vessel invasion to CNV site are detected, Bars = 50 µm (**D**) FACS analysis of single-cell suspension originated from retinas after 3 and 7 days of oral Lodamin treatment. Quantification of macrophage infiltration in mouse retinas originated from Lodamin treated (30 mg/kg, daily, oral) or mice which were treated with equivalent dose of vehicle. Macrophage population was detected as a double positive CD45+ and F4/80+ staining.

**Figure 4:**
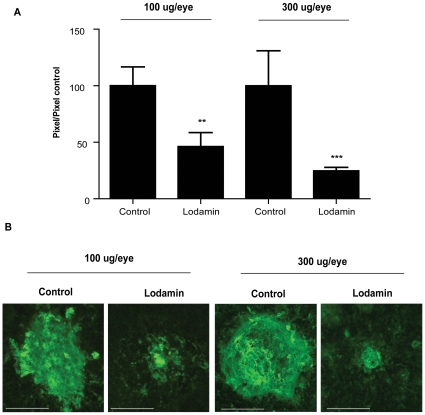
Lodamin injected intravitreal suppresses CNV progression. (**A**) CNV lesions were induced by a diode pumped solid state laser around the optic nerve through a slit lamp delivery system. Only lesions in which a subretinal bubble or focal serous detachment of the retina developed were used for the experiments. Four burns were performed per eye while leaving a space around the optic disc. At the day of laser induction intravitreal injection of Lodamin was performed. Size of CNV lesions after intravitreal injections of Lodamin: 100 µg Lodamin per eye (12.5 µg TNP-470 equivalent) or 300 µg Lodamin per eye. Data is presented as mean pixel number ± SEM (n = 7–17, *P<0.05, U-test). (**B**) Representative images of retinal flat-mounts stained with a lectin-FITC, Bars = 10 µm.

The effect of Lodamin on CNV progression in mice was determined after oral or intravitreal administration. Oral Lodamin was administered at multiple doses and durations: 15 mg/kg/day (for 7 days and 14 days of treatment) and 30 mg/kg dose (7 days). CNV lesions in choroidal flat-mounts were evaluated after lectin staining for blood vessels with anti-CD31 antibody. CNV area was significantly reduced by Lodamin in all treatment groups (Figure 3A). Higher doses and longer treatment led to an enhanced effect: 14 day treatment with 15 mg/kg q.d. gave 70.6% inhibition, while 7 day treatment at the same dose yielded 27.2% inhibition. Additionally, at 30 mg/kg/day dosing a more dramatic inhibition was obtained (50.5%) after 7 days. Figure 3A summarizes CNV area of all treatment groups. Representative images of retinal flat-mounts stained for blood vessels are shown in Figure 3B. H&E staining revealed that Lodamin substantially reduced the formation of fibrovascular response from the choroid which was induced in the spot rupture of Bruch's membrane by the photocoagulation laser (Figure 3C). Recruitment of macrophages into CNV lesions over time was evaluated by FACS in single-cell suspensions originating from mouse retinal tissue. Retinal infiltrated macrophages were reduced following Lodamin treatment (30 mg/kg/day, oral) as shown by the control group which had 2% and 3.6% infiltrating macrophages on day 3 and 7 respectively compared with Lodamin treated groups that showed 1.8% and 2.3% macrophages on days 3 and 7, respectively (Figure 3D). When Lodamin was introduced by a single intravitreal injection of 100 µg/eye or 300 µg/eye, 56% or 75% suppression were obtained, respectively, after 14 days (Figure 4). No significant difference was found when injecting PBS compared to vehicle (data not shown). Figure 4B shows representative CNV lesions from the different groups and their size measurements.

### Lodamin regresses established CNV lesions whereas sFlk-1 solely inhibits further growth

**Figure 5:**
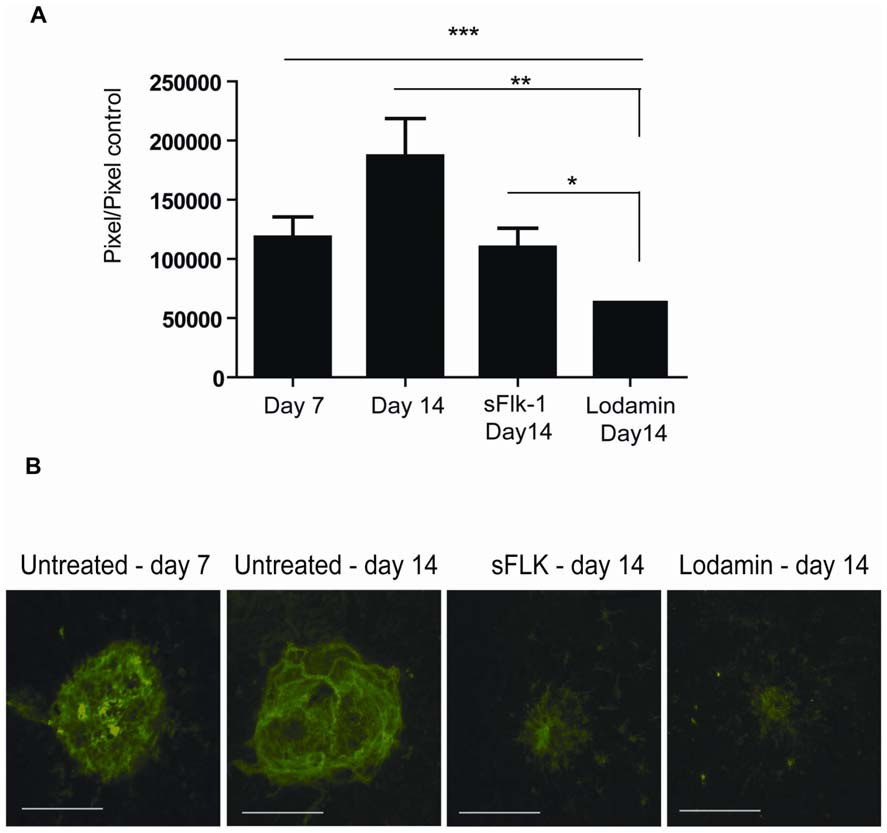
Comparison of the effect of Lodamin or sFlk-1 on choroidal vessel regression. For CNV regression studies intravitreal injections of Lodamin or sFlk-1, a recombinant mouse VEGF R2/KDR Flk-1 Fc Chimera, were performed on established CNV lesions at day 7 post laser induction. After 14 days of laser induction (7 days post intravitreal injection of Lodamin or sFlk-1) fluorescent images of choroidal flat-mounts were captured and CNV area (presented in µm^2^) in choroidal flat mount was evaluated using Scion image software. (**A**) CNV size data of untreated mice bearing laser induced CNV at day 7 and 14, compared to Lodamin or sFlk-1 treated mice 7 days after their injection. Data is presented as mean pixel number ± SEM (n = 15–27 spots, *P<0.05, U-test).

To determine whether therapies cause regression in CNV lesions or merely inhibit their further growth, we compared the effects of Lodamin to sFlk-1 on established CNV lesions induced by laser in mice. Seven days post laser induction, mice were injected intravitreally with Lodamin (100 µg/eye), or with sFlk-1 (3 µg/eye) or saline (as sham injection control). On day 14 post laser induction (7 days post treatment) the sizes of CNV in all mice groups were measured, and also compared to original size of the CNV in mice 7 days post laser induction (untreated). The Lodamin treated group was found to have CNV which were smaller by 65% compared to the sham group on day 14 (7 days post treatment). Importantly, when compared with the baseline size of CNV in untreated mice at day 7, a 45% regression was detected. While sFlk- treated group was found to have CNV which were smaller by 41% compared to the sham group on day 14, no significant reduction was found when compared to untreated mice at day 7, confirming inhibition of CNV growth without regression (Figure 5).

### Lodamin has minimal toxic effects on retinal function

**Figure 6:**
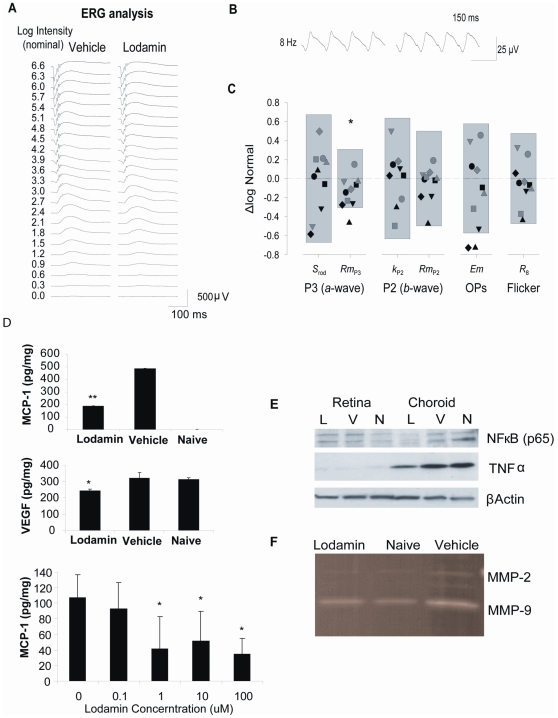
Retinal ERG and protein analysis post Lodamin treatments. Effects of intravitreal Lodamin on retinal function. (**A**) Representative full-field flash ERG responses from the eyes of mice 14 days after intra-ocular injection of vehicle (*left*) or 300µg /eye Lodamin (*right*). (**B**) Representative responses to 8 Hz flicker (**C**) The 5^th^ to 95^th^ prediction limits for ERG parameters in vehicle treated rats are shown; the dashed line represents the normal mean of all the paramters. Symbols are parameters values of individual Lodamin-treated mice, and represent the same mouse in each column. Data are ΔLogNormal (*eq. 3*). Only the saturating amplitude of the rod photoresponse (*Rm*_P3_) differed significantly after Lodamin. (**D**) Effect of oral Lodamin treatment on protein levels of pro-angiogenic and pro-inflammatory factors. (**D**) Levels of MCP-1 and VEGF extracted from whole eye tissues from naïve mice, mice treated with Lodamin (30mg/kg, oral every day for 7 d) or vehicle (same equivalent amount of mPEG-PLA micelles). Data of ELISA assay are presented as a mean of pg/ml concentration ± SD (n = 6, * P<0.05, t-test). Bottom: ELISA quantification of secreted MCP-1 from RPE to the medium post 24 h incubation with Lodamin at different doses (0.1–100 nM TNP-470 equivalent). (**E**) Western blot protein analysis of samples of tissue lysis of retina or choroid of the different treatment groups: N = naïve mice, V = vehicle treated mice, L = Lodamin treated mice. Factors detected are TNFα (26 kDa) (**F**) Zymogram showing MMP-2 and MMP-9 activity in ocular tissue following oral Lodamin treatment of mice (30 mg/kg/day 7 days).

To evaluate possible adverse side effects of Lodamin treatment, retinal function was assessed by electroretinography (ERG). Normal C57BL/6J mice were injected intravitreally with the highest dose studied of Lodamin (300 µg; *n* = 10) or vehicle (*n* = 10) and tested 14 days after injection. Responses to a ∼6.6 log unit range of intensities of full-field flashes presented to the dark-adapted eye were recorded (Figure 6A) as was the response to an 8 Hz flickering light presented in the presence of an adapting background (Figure 6B). Rod photoreceptor response sensitivity (*S*_rod_) and saturating amplitude (*Rm*_P3_) were derived from the ERG *a*-wave and postreceptor sensitivity (*k*_P2_) and saturating amplitude (*Rm*_P2_) were derived from the ERG *b*-wave (Figure 6C). The saturating energy in the ERG oscillatory potentials (*Em*), and the trough-to-peak amplitude of the flicker response (*R_8_*) were also determined (Figure S2A, S2B). For each of the six ERG parameters, in log scale, the mean and 5^th^ and 95^th^ prediction limits for normal were calculated and compared to those of Lodamin-treated mice (Figure 4C); Nearly every observation (56 of 60) in Lodamin-treated mice fell within the normal range. Only *Rm*_P3_ differed significantly (*P* = 0.46) between groups, by <0.15 log unit. In histological examination no toxicity was observed in histological H&E whole eye samples (Figure S2C).

### Lodamin reduces pro-angiogenic and inflammatory factors in CNV bearing mice

Levels of different pro-inflammatory and pro-angiogenic factors were measured using specific antibody by enzyme-linked immunosorbent assay (ELISA) or western blot analysis. Significant reductions of MCP-1 and VEGF in retina-choroids tissues were found in Lodamin treated mice by ELISA. MCP-1 was not detectable in naïve mice but was found in high concentrations in CNV bearing mice (486±3 pg/mg in control CNV mice) and 61% lower level in Lodamin treated mice (Figure 6D). Unlike MCP-1, VEGF baseline levels in naïve mouse eyes were almost as high as these in CNV bearing mice (313 pg/mg, 320 pg/mg respectively). Lodamin treated mice reduced VEGF levels to 24% below the baseline (243 pg/mg). MCP-1 levels in primary RPE cultures were also measured by ELISA and were found to be reduced by Lodamin in a dose dependant manner after 24 h treatment (39%, 55%, 83% reduction was obtained with 1, 10, 100 nM Lodamin, respectively). All ELISA data were normalized to total protein in each sample (Figure 6D).

The levels of TNFα, a major inflammatory cytokine, were higher in choroid than in retina and were reduced in the choroid by Lodamin (Figure 6E). NF-κB, a pro-inflammatory transcription factor induced by TNFα, was detected in both retina and choroid and was also reduced in the choroid by Lodamin. MMP-2 and 9 activity in CNV lesions was determined following oral Lodamin treatment (30 mg/kg q.d for 7 days) using zymogram (Figure 6F). The enzymatic activity was accelerated in tissues with CNV compare to naïve mouse tissues, while both MMP-2 and 9 activities were reduced by Lodamin to the levels of naïve mice.

### Lodamin reduces MCP-1 and VEGF induced vessel permeability

**Figure 7:**
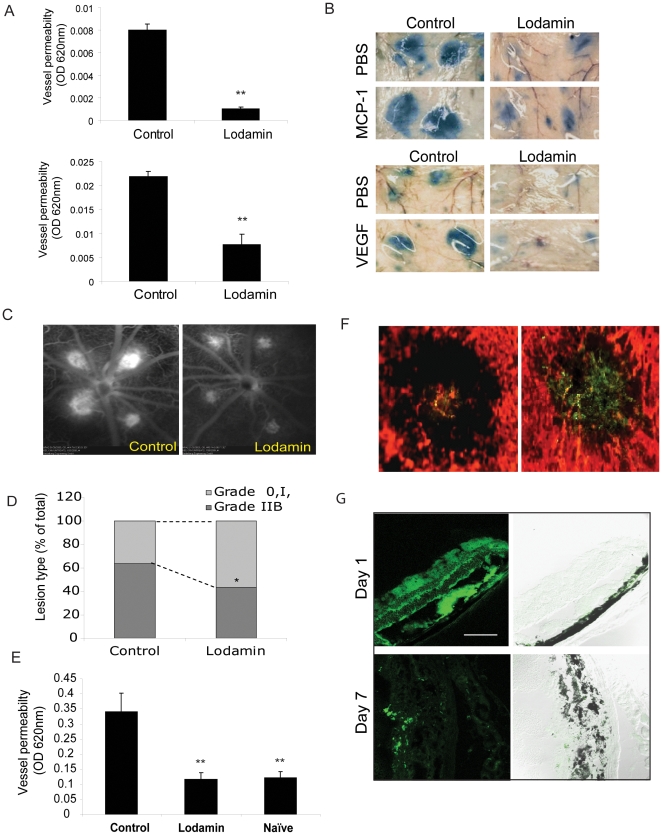
Lodamin reduces vessel permeability in the modified Miles assay and in retinal angiography. (**A**) Left: quantification of modified Miles assays performed in mice after induction of vessel permeability using MCP-1 (50 pg) and VEGF (50 ng); extracted dye contents were quantified by measuring at 620 nm. Data are expressed as mean ± SEM (n = 10, *P<0.05, t-test). (**B**) Representative photos of mouse skin showing diminished dye in mice treated with oral Lodamin 1 and 3 h before conducting the assay. MCP-1 induces vessel permeability in a dose dependent manner, already at 10 pg/ml, MCP-1 induced significant vessel leak compared with PBS. (**C**) Fluorescein angiography of CNV lesions of rats treated with Lodamin or vehicle. Fluorescein angiography was performed at day 7 after laser photocoagulation and Lodamin was administered 1 and 3 h before performing the imaging. (**D**) The percentage of lesions graded as 0, I, IIA, defined as no leakage to moderate leakage, and IIB, considered clinically relevant leakage, in vehicle-treated (n = 6 eyes). A significant reduction of vessel leak was observed after an acute treatment of Lodamin. (**E**) Lodamin reduces whole eye vessel permeability as determined by Evan's blue extraction method. Mouse eyes treated with Lodamin (3 h before conducting the assay) were 65% less than the control. Data are presented as mean ± SD (n = 5, *P<0.05, t-test). (**F**) Accumulation of 6-coumarin labeled Lodamin injected i.v at the site of CNV lesion in mouse compared to an injection of free 6-coumarin. In red: blood vessels stained with rhodamine concanavalin A, in green: 6-coumarin dye. Bar = 20 µm. (**G**) Bioditribution of 6-coumarin labeled Lodamin (green) in mouse retina side sections 1 day or 7 days post intravitreal injection. 10 µm Section were imaged by confocal microscopy using optical sections with 488-nm argon, diode lasers. Bar = 20 µm.

We further investigated MCP-1's role on vessel permeability and Lodamin's ability to antagonize this effect. In the modified *in vivo* Miles assay, MCP-1 enhanced vessel permeability in concentrations of 10, 60, 100, 250 pg/ml (n = 15). Representative mouse skin patches showing Evan's blue leakage in MCP-1 injection spots are presented in Figure S3. The effect of Lodamin on MCP-1-induced vessel leakage was measured after 5 days of oral pretreatment with 30 mg/kg/day in C57Bl/6J mice (Figure 7A). An 87% reduction in vessel permeability was obtained. Similarly, a Miles assay using VEGF was performed and demonstrated a 65% reduction of acute vascular leakage following the same oral Lodamin treatment for 5 days (Figure 7B).

### Lodamin reduces ocular vascular permeability

In order to evaluate the effect of Lodamin on ocular vascular leakage while excluding its direct angiogenesis inhibitory effects, CNV lesions of the same stage were used to compare vessel leakage in mice and in rats. Laser-induced CNV in rat retinas were imaged by fluorescein angiography (n = 6). Rats with established CNV lesions (7 days post laser injury) were treated with a single Lodamin treatment 3 h prior to quantifying leakage (60 mg/kg). The lesions were graded using angiography and showed a significant reduction of retinal vessel permeability (Figure 7 C,D). A modified VEGF Miles assay was also performed to evaluate Lodamin's effect on ocular vessel permeability in mice with an established CNV lesion. Similarly, when Lodamin (60mg/kg, n = 5) was administered once 3 h before Evans blue injection, 65% reduction in vessel leakage was obtained by this short-term Lodamin treatment (Figure 7 E).

### Polymeric micelles accumulate in CNV site

The biodistribution of fluorescent-labeled Lodamin in CNV lesions was determined 3h post tail vein injection in C57Bl/6J mice. A model fluorescent hydrophobic small molecule (6-coumarin, MW = 350 gr/mole, Sigma) was used to determine differences between accumulations of the free molecule and its encapsulated form. Compared with the free molecule, labeled polymeric micelles were detected in greater extent in CNV sites confirming passive targeting (Figure 7F, n = 3). In a separate experiment we used the same labeled micelles as a model to study Lodamin's distribution in the retina using retinal cross section in mice. Micelles were injected intravitreally on the day of laser induction and was imaged using retinal cross sections 1 and 7 days post injection (Figure 7G).

## Methods

Mice (C57Bl/6J, 8 weeks old) were purchased from Jackson Laboratories (Bar Harbor, ME, USA). For rat studies, non pigmented Lewis rats (8–10 weeks old, Charles River Laboratories) were used. All protocols were approved by the Institutional Animal Care and Use Committee at Children's Hospital Boston and were conducted in accordance with the Association for Research in Vision and Ophthalmology's Statement for the Use of Animals in Ophthalmic and Vision Research. (Protocols no. A09-10-1531 and A08-08-1208R CHB, 07-09-009, MEEI)

### Cell culture

RPE cells were cultivated as has been previously described[11]. As determined by flow cytometry, the primary RPE cultures were found to be more than 98% cytokeratin positive (Clone PCK-26, Sigma). HMVECs, were purchased from Cambrex Bio Science Inc. (Rockland, ME) and were maintained on collagen I coated plates with endothelial cell growth medium containing 2% FBS (EGM-2; Cambrex, Inc.) at 37°C with humidified 95% air / 5% CO_2_.

### Preparation of Lodamin and florescent labeled micelles

Lodamin was prepared as previously described[10]. Briefly, TNP-470 (D. Figg) was conjugated to a diblock co-polymer mPEG-PLA using a two-step reaction. In the first step succinated mPEG2000-PLA1000 with free carboxic acid end-groups (Advanced Polymers Materials) was reacted with ethylenediamine (Sigma-Aldrich) by using ethyl(diethylaminopropyl) carbodiimide (EDC) and a catalyst N-hydroxysuccinimide (NHS). In the second step, the amine-containing polymer was mixed with TNP-470 (350 g), dissolved in DMSO and the solution was stirred for 4 h at 25°C. The polymeric micelles were formed by dialyzing the DMSO solution of the conjugate against double distilled water using a regenerated cellulose dialysis bag (MWCO 1000, Spectra/Por Biotech Regenerated Cellulose, VWR). The micelles were then lyophilized and stored at −20°C in a dry environment until use. For fluorescent labeled Lodamin, a commonly used hydrophobic marker 6-coumarin (Sigma-Aldrich) at 0.1% wt/wt was added to the polymeric solution before the final dialysis step. The doses of Lodamin as noted are presented as TNP-470 equivalent.

### *In vivo* Matrigel angiogenesis assay

Briefly, C57BL/6J mice were inoculated subcutaneously with growth factor-reduced Matrigel Matrix (BD Bioscience) mixed with recombinant human VEGF (10 ng/ml) and bFGF (10 ng/ml) (R&D Systems). Mice were treated with daily oral Lodamin for 7 day (15 mg/kg) or with the equivalent dose of vehicle (empty micelles). After 7 days, Matrigel plugs were removed, embedded in OCT medium (OCT, Tissue-Teck, USA) and immunohistochemistry was performed using Vectastain Elite ABC kit (Vector Laboratories) followed by anti-CD31 reaction with for microvessel staining. Detection was carried out using a 3,3*¢*-diaminobenzidine chromogen (DAB), which results in a positive brown staining. Quantification of endothelial cells and macrophages in Matrigel plugs was performed by FACS following enzymatic digestion of Matrigel plugs as described elsewhere [12]. Immunostaining was performed in the presence of rat anti-mouse F4/80-PE and CD45-APC. Flow cytometry was performed using FACS Calibur and CellQuest software (BD Biosciences, San Jose, CA, USA). Endothelial cells were defined as CD31+CD45− and macrophages were defined as F4/80^+^ CD45^+^ cells.

### Corneal micropocket assay

The corneal micropocket angiogenesis assay was performed as previously detailed[13]. Pellets containing 80 ng carrier-free recombinant human bFGF (R&D Systems, Minneapolis, MN) were implanted into micropockets created in the cornea of anesthetized mice. Mice were treated daily with 15 mg/kg Lodamin for 5 days, and then the vascular growth area was measured using a slit lamp. Photos of mouse eyes were taken on day 5 of treatment. The area of neovascularization was calculated as vessel area by the product of vessel length measured from the limbus and clock hours around the cornea, using the following equation: Vessel area (mm^2^) = (π×clock hours×vessel length (mm)×0.2 mm].

### DTH reactions

DTH reactions were induced in the skin of 8-week-old C57Bl/6J male mice as previously described [14]. Mice were sensitized by topical application of 2% oxazolone solution in vehicle (acetone: olive oil, 4∶1 v/v) to the shaved abdomen (50 µl) and to each paw (5 µl). After 5 days, right ears were challenged by topical application of 10 µl of a 1% oxazolone solution, and the left ears were treated with vehicle alone. On the same day mice were treated with Lodamin (15 mg/kg orally) and ear thickness was then measured daily (3 measurements per ear using a caliper) for 12 days. On day 12, 3 mice from each experimental group were euthanized and their ears were removed, fixed in 10% formalin and processed for H&E-stained paraffin sections and for immunohistochemical staining. Specific antibodies for detecting blood vessels (anti-CD31), macrophages (anti-F4/80) and monocytes (anti-CD45) were used and avidin/biotin complex (ABC; Vecstatin Elite. ABC kit; Vector Laboratories) followed by DAB staining were performed obtaining a positive brown staining.

### Induction of CNV and flat-mount preparation

Laser-induced CNV was generated by a previously described technique with some modifications [15]. C57Bl/6J mice were anesthetized with an intraperitoneal administration of avertin (400 mg/kg). A mixture of 0.5% tropicamide and 0.5% phenylephrine hydrochloride (Mydrin P; Santen Pharmaceutical, Osaka, Japan) was applied to both eyes to dilate the pupils. Lesions were induced by a diode pumped solid state laser (0.1 s; spot size, 50 µm; power 150 mW) around the optic nerve through a slit lamp delivery system using a Nidek photocoagulator (GYC2000, Nidek, Osaka, Japan) while a hand-held cover slide was used as a contact lens. Only lesions in which a subretinal bubble or focal serous detachment of the retina developed were used for the experiments. For this purpose, four burns were performed per eye while leaving a space around the optic disc. Lodamin treatments (oral or intravitreal) were started at the day of CNV induction (day 0) and the sizes of CNV lesions were determined after 7 or 14 days. For regression studies the intravitreal injections of Lodamin or sFlk-1 were performed at day 7 post laser induction. After 7 or 14 days of laser induction, mice were euthanized and their eyes were removed and fixed in 4% paraformaldehyde for 60 min. The cornea and lens were removed, and the entire retina was carefully dissected from the eyecup. Radial cuts (average, eight) were made from the edge of the eyecup to the equator, and then washed with cold buffer (0.3% Tween 20) in PBS. Blood vessels were labeled using a 1∶200 dilution of isolectin IB4 conjugated with Alexa Fluor 488 (Invitrogen-Molecular Probes, Eugene, OR, USA) or lectin-FITC (Vector Laboratories). The eyecup was flat-mounted in an aqua-mount with the sclera facing down and the choroid facing up. Fluorescent images of choroidal flat-mounts were captured using a CCD camera (DC500, Leica, Switzerland). The CNV area (presented in µm^2^) in choroidal flat mount was evaluated using Scion image software.

### Analysis of cell population in retina by Flow cytometry

Single cell suspensions were prepared from C57BL/6J mouse retinas with CNV lesions. In order to collect a sufficient number of ocular-infiltrating cells for flow cytometry, 20 separate burns were delivered similar to the panretinal photocoagulation procedure in humans. The percent of macrophages in single-cell suspensions originated from mouse retinas (days 3 and 7 post laser procedure) was evaluated for Lodamin treated or untreated mice (3 days, oral, 30 mg/kg q.d). In the third day of treatment mice were euthanized, the eyes were enucleated and the anterior segment (cornea, iris, and lens) was removed. The posterior segment of the eye including sclera, choroid, and retina was disrupted with scissors and then incubated with 2.8 units/ml Liberase Blendzymes (Roche Indianapolis, IN, USA) at 37°C for 45 min. Digested tissue was then filtered through a 40 µM cell strainer and resuspended in FACS Buffer (PBS, 1%BSA, 5 mM EDTA/0.05% sodium azide). Immunostaining was performed in the presence of rat anti-mouse F4/80 PE and CD45-APC by incubating the cells for 30 min on ice. Flow cytometry was performed using FACS Calibur and CellQuest software (BD Biosciences, San Jose, CA, USA). Macrophages were defined as F4/80^+^ CD45^+^ cells. Preparations from two eyes were pooled in order to obtain a sufficient number of viable cells for a flow cytometric analysis in the above process. A total of four eyes (two individual pools) were examined per group (5 mice per group).

### *In vivo* Miles assay for vessel permeability

In order to determine whether MCP-1 affects vessel permeability, a Miles assay was performed in C57Bl/6J mice. Briefly, shaved mice were injected intravenously with 200 µl of 0.1% Evans blue dye (Sigma). After 10 min, different concentrations of MCP-1 were injected intradermaly (i.d.) in designated spots on the mouse back (5, 10, 50, 100, 250 pg/ml, 50µl per spot, 6 spots including PBS). To quantify the extent of leakage, equally sized (8 mm diameter) skin regions surrounding i.d. injection sites were removed 30 min after injection and placed in 1 ml of formamide at room temperature for 48 h allowing dye extraction. The optical absorption of the samples was measured at 620 nm using 96 well microplate reader Victor2 (PerkingElmer Life Siences, Boston, MA, USA). In a separate experiment, we determined the effect of Lodamin on vascular permeability *in-vivo* using standard Miles assay. For these experiments C57Bl/6J mice were treated for 5 days with oral Lodamin (30 mg/kg/day). PBS (50 µl) and carrier free VEGF (50 ng in 50 µl) or MCP-1 (R&D Systems, USA, 50 pg in 50 µl) were injected i.d. in both left and right flanks as well as at single or dual dorsal sites 10 min post Evans blue injection. The extent of leakage was calculated by comparison with PBS-treated controls.

### Laser induced retinal vascular permeability

In order to specifically evaluate the effect of Lodamin on laser-induced retinal leakage while excluding its anti-angiogenic activity, CNV lesions of the same size (same day post laser injury) were used to compare blood vessel permeability in mice and rats. Two different assays were performed: modified Miles assay in mice and angiography in rats. The modified Miles assay was performed similarly to the standard procedure: in brief, CNV was induced by laser beam (4 spots) as detailed above. Seven days post laser procedure mice were treated with Lodamin (60 mg/kg) 3 h prior to intra-orbital injection of Evan's blue (100 ul, 0.1% in saline, Sigma). After 10 min, mice were sacrificed, eyes were removed and washed 3 times with PBS and Evan's blue dye was extracted using Formamide (0.5 ml/sample) for 48 h at 37°C. Two controls were used: untreated CNV-bearing mice or naive mice.

Angiography was performed on rats using Fluorescein imaging. In brief, 7 days post laser injury vascular leakage of CNV lesions were assessed using fluorescein angiography (FA) as previously described [16]. Briefly, FA was performed on anesthetized rats from Lodamin treated or untreated groups, using a scanning laser ophthalmoscope (SLO; HRA2; Heidelberg Engineering, Dossenheim, Germany). Fluorescein injections were administrated intravenously (0.2 ml of 2% fluorescein; Akorn, Decatur,IL, USA). The grading criterion was as follows: grade-0 lesions had no hyperfluorescence, grade-I lesions exhibited hyperfluorescence without leakage, grade-IIA lesions exhibited hyperfluorescence in the early or midtransit images and late leakage. Grade-IIB lesions showed bright hyperfluorescence in the transit images and late leakage beyond the treated areas, Grade-IIB lesions were defined as clinically significant.

### Biodistribution

In order to evaluate the accumulation of Lodamin in its micelle structure at the CNV lesion site, a comparison between free or encapsulated fluorescent marker was performed. Lodamin labeled with 6-coumarin (1 mg/kg) was injected into the retro-orbital plexus of mice while an equivalent dose of free 6-coumarin (in ethanol) was injected as control. After 3 h cornea and lens were removed, and the entire retina was carefully dissected from the eyecup. Radial cuts were made from the edge of the eyecup to the equator, and then washed with PBS. Blood vessels were labeled using a 1∶50 dilution of rhodamine concanavalin A (RL-1002, Vector Laboratories, Burlingame, CA, USA). The eyecup was flat-mounted in an aqua-mount with the sclera facing down and the choroid facing up. Fluorescent images of choroidal flat-mounts were captured using OpenLab software version 2.2.5 (Improvision Inc.) with standardized illumination and contrast. In order to evaluate ocular distribution and drug stability we performed a separate experiment in which 6-coumarin labeled Lodamin was injected intravitreally into mice (100 µg/eye). After 1 day or 7 days post laser induction and drug injection, eyes were removed and a serial of side sections (10 µm) were taken to detect the CNV lesion site. The distribution of labeled Lodamin was detected by confocal 1 and 7 days post injection in histological side sections of mouse eyes. Sections were imaged by confocal microscopy using optical sections with 488-nm argon, diode lasers. Bar = 20 µm.

### Antibodies and materials

We used the following specific antibodies for western blot: anti mouse TNFα (abcam, # ab6671), anti- NFκB p65 (SantaCruz Biotechnology, # sc-109), HRP-anti-actin (Sigma, #A3854). For FACS analysis we used PE-F4/80, APC-CD45 and PE-CD31 purchased from BD bioscience. For immunohistochemistry we used anti-CD31, anti-CD45, anti-F4/80 antibodies from BD Bioscience. Recombinant mouse VEGF R2/KDR/Flk-1 Fc Chimera (sFlk-1) was purchase from R&D Systems. General reagent was obtained from Sigma-Aldrich unless noted otherwise.

### Zymography

Gelatin zymography was used to examine levels of MMP-9 and MMP-2 in the samples. Equal amounts of protein (50 ug) were mixed with sample buffer and resolved via electrophoresis using 10% Ready Gel Zymogram Precast Gels with gelatin (BioRad Laboratories, Hercules, CA). After electrophoresis, gels were soaked in Zymogram Renaturing Buffer (BioRad Laboratories, Hercules, CA) for 30 minutes with gentle agitation at room temperature. The gels were then rinsed and incubated overnight at 37°C in Zymogram Developing Buffer (BioRad Laboratories, Hercules, CA). Later, the gels were stained for 15–30 min in 0.5% Coomassie Blue R-250 in acetic acid, isopropyl alcohol, and water (1∶3∶6), distained for 1 h in acetic acid, ethanol, and water (1∶3∶6), and then photographed. The enzyme activity was detected as clear bands on uniform blue stained background. Recombinant mouse MMP-9 and MMP-2 (R&D Systems, Minneapolis, MN) were used as positive control.

### Protein analysis

To measure protein level in ocular tissue, an equal number of mouse retinas and choroids isolated from the different treatment groups were lysed in RIPA buffer (n = 5). CNV bearing mice were burned with high energy laser in 20 spots to enhance the immune reaction. The total protein level in the tissue samples was determined using microBCA kit (Pierce, Rockford IL ,USA) and an equal amount of 50 µg total protein was mixed with ×6 sample buffer (2% SDS, 60 mM Tris, HCl pH 6.8, 0.02% DTT). The samples were then separated in 4–20% SDS-PAGE and transferred to a Polyvinylidene Fluoride (PVDF) membrane (BioRad). Reaction of Horseradish peroxidase (HRP)-conjugated secondary antibodies against specific primary antibodies followed by ECL development (Amersham), and autoradiography (Kodak films) were performed. Similar protocol was used for cell-lyses protein analysis. To measure murine VEGF and MCP-1 levels in tissues, standard ELISA kits were used following manufacturer instruction (R&D systems).

### Electroretinography

Dark-adapted full-field ERGs were obtained as previously described [17]. By fitting the Hood and Birch formulation [18] of the Lamb and Puch model [19], [20] of phototransduction to the a-wave, we obtained the photoreceptor's kinetics (Srod) and saturated amplitude (RmP3). From the response vs. intensity relationship of P2, the putatively-pure postreceptor response derived from the *b*-wave we determined bipolar sensitivity (log kP2) and saturated amplitude (RmP2). The oscillatory potentials (OPs) obtained after passing P2 through a 5th-order Butterworth filter (bandpass 50–250 Hz) were evaluated in the frequency domain [21] to estimate maximal OP energy (Em). Finally, the retinal response to light-adapted 8 Hz flicker was measured (R8). Prior to ERG testing, mice were adapted to dark overnight. All preparations were made under dim, red illumination as previously described [17] for the rat, except that the electrode was fitted to the eye of the mouse. We delivered stimuli and collected responses using an Espion *e^2^* with ColorDome Ganzfeld stimulator (Diagnosys LLC, Lowell, MA). The timing and intensity of the stimuli were fully under computer control and thus did not differ between subjects. Signals were amplified in the frequency range of 0.3–1,000 Hz and sampled at a minimum of 2,000 Hz. Body temperature was maintained with a heating pad. Flash intensity is reported in nominal units.

Photoreceptor function were evaluated by ensemble fitting the Hood and Birch model[18]of the activation of phototransduction to the leading edge of the ERG *a*-waves elicited by bright, xenon arc flashes which saturated the rod photocurrent. The model takes flash intensity (*i*) and elapsed time (*t*) as its inputs, such that P3(*i*, *t*) = (1−exp(−½•*S*_rod_•*i*•(*t*−*t*_d_)^2^))•*Rm*_P3_ for *t*_d_<*t*<10 ms, (*eq. 1*) where *S*_rod_ summarizes the kinetics of the series of processes initiated by the photoisomerization of rhodopsin and resulting in closure of the channels in the plasma membrane of the photoreceptor, *t*_d_ is a brief delay, and *Rm*_P3_ is the saturated amplitude of the photoreceptor response (µV). *S*_rod_ is related to the amplification constant (*A*) of Lamb and Pugh[19], [20] and *Rm*_P3_ is proportional to the magnitude of the dark current[22].To evaluate postreceptor retinal function, a series of ‘green’ LED flashes were presented, from one eliciting a small (<20 µV) *b*-wave to one saturating the rod bipolar cells' response, and derived the putatively pure postreceptor response, P2, was derived by digitally subtracting the derived photoreceptor response, P3 (*eq. 1*), from each intact ERG. We obtained objective estimates of P2 amplitude by fitting of an inverted gamma function to the derived responses[23]. To determine the maximal amplitude of the rod bipolar cell response (*Rm*_P2_), we fit the Michaelis-Menton equation, *f*(*i*) = *a*•*i^b^*/(*i^b^*+*c^b^*) *eq. 2* to the response *vs.* intensity relationship of P2, where *f*(*i*) is the amplitude (µV) of the bipolar cell response (P2) to a stimulus of *i* intensity (*R*_P2_), *a* (µV) is defined as *Rm*_P2_, and *c* is the is the flash intensity that elicits a P2 response with half-maximal amplitude, defined as *k*_P2_; the exponent, *b*, was fixed at unity. Log *k*_P2_ was taken as a measure of post-receptor sensitivity.

To demonstrate the electroretinographic oscillatory potentials (OPs), we passed the derived P2 responses through a digital, 5^th^-order Butterworth filter (MATLAB; The MathWorks, Natick, MA) with band-pass 50 to 250 Hz; the resulting OPs were evaluated in the frequency domain as previously described[21]. In brief, we subjected the first 256 samples (128 ms) of each record to discreet Fourier transform (radix-2 FFT algorithm; MATLAB) to produce a power spectrum. Then, to characterize the OPs, we fit the data by a Gaussian, the integral of which we took as proportional to OP energy (joules). We obtained an estimate of maximal OP energy, *Em*, by fitting *eq. 2* such that *f*(*i*) is the energy of the OPs in the response to a stimulus of *i* intensity, *a* is defined as *Em*, and, *c* is the flash intensity which elicits OPs with half-maximal energy; *b* was free to vary.

A cone-mediated retinal response was elicited by presenting 8 Hz ‘green’ square-wave flicker in the presence of an ‘amber’ background that suppressed the saturating, dark adapted *a*-wave by >80%. We measured the trough-to-peak amplitude of the flicker response, *R*_8_. We expressed ERG data as ΔLogNormal: 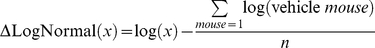 *eq. 3* where *x* an individual observation and *n* is the number of vehicle-treated mice (10). By expressing the data in log values, changes in observations of fixed proportion, either up or down, become linear, consistent with a constant fraction for physiologically meaningful changes in parameter values [17].

### Analysis of cell population in retina by flow cytometry

Single cell suspensions were prepared from C57BL/6J mouse retinas with CNV lesions. In order to collect a sufficient number of ocular-infiltrating cells for flow cytometry, 20 separate burns were delivered similar to the panretinal photocoagulation procedure in humans. The quantity of macrophages in single-cell suspensions (originated from mouse retinas from days 3 and 7 post laser procedure) was evaluated for Lodamin treated (oral, 30 mg/kg q.d for 3 days), vehicle or untreated mice. In the third day of treatment mice were euthanized and retinas analyzed.

### Statistical Analysis

For CNV and retinal permeability studies a Mann–Whitney U-test was used using PrisM software. For the other studies an unpaired, two-tailed Student's *t*-test was used when comparing between the different groups. The significance level (α) for all tests was *p*<0.05. For ERG studies the statistical analysis is detailed in the methods.

## Discussion

Significant progress has been made towards the elucidation of the pathophysiology of angiogenesis and the role of antiangiogenic therapies in the treatment of pathological neovascularization [24]. This has accelerated the development of antiangiogenic therapies for neovascular AMD and drug development for other retinal pathologies [24]. To date inhibition of angiogenesis, both directly and indirectly, is one of the leading therapeutic approaches in neovascular AMD.

However, ocular drug-delivery faces many challenges for new drugs, particularly for lipophilic compounds that cannot be introduced directly in aqueous solutions. Many small-molecule angiogenic inhibitors, such as TNP-470, have poor water solubility necessitating the development of unique formulations to facilitate their use. For these drugs, particulate formulations can offer improved ocular drug delivery since they are usually more stable than other colloidal systems. Lodamin comprises many of the requirements for advanced ocular delivery: it can be introduced into an aqueous solution, is nontoxic, biocompatible, biodegradable, and has slow-release properties[10]. This is the first report of Lodamin as treatment for retinal neovascular disorders.

Lodamin was found to be significantly effective in suppressing laser induced CNV in mice, both by oral daily administration and by single intravitreal injection, where up to 75% inhibition was obtained using only a single injection. In fact, its activity was able to promote regression of the CNV lesions by 45% post a single intravitreal injection. This ability of Lodamin was particularly important considering the fact that VEGF inhibitors can reduce ocular edema but not regress existing CNV. In our experiments, we confirmed that a potent VEGF inhibitor, sFlk-1, inhibited CNV growth but could not regress the vessels once established. The stronger activity of Lodamin can be explained by its broad mechanism of action combined with its high stability and slow release properties. In addition, these characteristics reduce the need for repeated, frequent injections required in order to achieve therapeutic outcomes, as currently exist with VEGF blockers.

Our data demonstrated Lodamin's suppressive effects on vascular permeability and inflammation, in addition to its direct effect on angiogenesis. This can be partly explained by our findings that the treatment led to a substantial reduction of secreted MCP-1 protein level in RPE cells and in CNV lesions in mice. RPE cells are responsible for generating and maintaining the extracellular and photoreceptor matrices, as well as the integrity of Bruch's membrane. Impaired function of these cells can cause lipid and protein accumulation in the vicinity of Bruch's membrane and result in formation of drusen that is commonly found in AMD patients [25]. RPE cells secrete chemoattractants and various pro-inflammatory cytokines such as interleukin-8 (IL-8), MCP-1 and TNFα. MCP-1, a small cytokine of the C-C chemokine family, is a potent chemoattractant for neutrophils and macrophages which stimulatates angiogenesis and vascular leak, further contributing to AMD progression [26]–[27]. Elevated MCP-1 levels have been found in the vitreous of patients with retinal neovascularization [26], [28]. Moreover, MCP-1 has been associated with other systemic inflammatory and autoimmune diseases such as diabetes and proliferative vitreoretinal disorders [29], [30]. In support of these observations, our *in vivo* data showed that Lodamin suppressed MCP-1 production at the CNV site and this was accompanied with a reduction of infiltrating macrophages in the retina.

Interestingly, we found that Lodmain could antagonize MCP-1 induced vascular leak in a dose dependant manner as potently as when VEGF was used as the inducer of vascular permeability. These results can explain the significant reduction that was found in retinal edema following angiography or Evan's blue extraction, and also the diminished tissue edema and spongiosis found in the DTH reaction. It should be noted that the effect of the drug on retinal edema was very rapid, as only a single oral pretreatment 3 h prior to angiography was sufficient to substantially reduce of vessel permeability.

The reduction of MCP-1 levels by Lodamin was found to be associated with a diminution of the transcription factor nuclear factor k-B (NFκB) in the choroids of Lodamin treated mice. NFκB regulates the production of MCP-1 (which is stimulated by TNF-α and IL-6) and is known to be a key payer in the immune response, cancer, inflammatory and autoimmune diseases. [31]. Lodamin suppressed other pro-angiogenic and pro-inflammatory factors including VEGF, TNFα and matrix metalloproteinases (MMPs) at the CNV site, effectively preventing the progression of ocular neovascularization.

The gold standard treatment of neovascular, or wet, AMD is primarily based on VEGF targeting, despite the critical role of other cytokines and growth factors such as MCP-1, TNF α and basic fibroblast growth factor (bFGF) in AMD pathology [4]. Because Lodamin can target broader molecular pathways, we expect it will improve therapeutic outcomes compared to VEGF blockers. Furthermore, the specific structure of Lodamin, a polymeric nanomicelle, enhances its accumulation in CNV lesions compared to the injection of free small molecule (as seen in Figure 7F) suggesting ideal biodistribution via systemic administration. This effect may be due to the Enhanced Permeability and Retention effect (EPR)[32] caused by the leaky vasculature in neovascular sites. Importantly, despite the broad spectrum activity of Lodamin, it was well tolerated and showed minimal toxicity in the retina as indicated by histological analysis and ERG. Further studies are required to determine the effects following chronic use. Lastly, the slow release properties of Lodamin suggested that the frequency of treatments can be reduced. This is particularly important in the case of intravitreal injection, which is more problematic for patients, requires clinician expertise, and is associated with some risks.

Taken together, we maintain that broad spectrum antiangiogenic drugs such as Lodamin are important candidates for therapeutic treatment of ocular neovascular diseases in humans and can bring a major change in the approach to AMD treatment.

## Supporting Information

Figure S1 Lodamin inhibits angiogenesis and inflammatory response in Matrigel plug angiogenesis assay and DTH reaction. (A) Upper panel: representative Matrigel plugs exposed under mouse skin (bar = 1 cm). Vehicle treated mice presented bloody plugs surrounded by massive blood vessels, compared to Lodamin treated mice which had poor vasculature. bar = 100µm. Lower panel: FACS dot plots of endothelial cells (CD31+/CD45, marked in red squares) or macrophages (CD45+F4/80+, marked in red squares) from single cell suspension originated from Matrigel plugs. (B) Immunohistological analysis of mice ears post DTH reaction: staining of macrophages using F4/80 marker. Control ears exhibited an excessive inflammation. Bar = 50 µm. No obvious difference was found in macrophage distribution however, the total number of macrophages present in control mice was elevated due to greater amount of tissue, associated with increased swelling.
(9.63 MB TIF)

Figure S2 Evaluation of retinal toxicity post intravitreal injection of Lodamin (A)Retinal electroradiography post Lodamin intravitreal injection. The a-waves in response to the eight brightest flashes are replotted at increased gain. The black portions are fitted with the Hood and Birch model of the activation of phototransduction (eq. 1; colored lines). (B) Fits of eq. 2 to the response vs. intensity relationship of P2. Drop lines indicate the intensity that elicits a flash with half-maximum amplitude. (C) Histological cross sections of retinal tissues. Retinal tissues taken 14 days post intravitreal injection of Lodamin (300 µg/eye) were compared to naïve mouse retinas. Representative images are shown (Bars = 50 µm). No apparent retinal tissue changes were detected, both eyes presented normal structures of (1) choroid (2) photoreceptors (3) outer nuclear layer (4)outer plexiform layer (5) inner nuclear layer (6) inner plexiform layer and (7) ganglion cell layer. The total retinal thickness remained unchanged.
(5.11 MB TIF)

Figure S3 Induction of vessel permeability by MCP-1 demonstrated in Miles assay. Representative photos of mouse skin showing dose response MCP-1-induced vessel permeability. Graph represent the quantification of Miles assays performed in mice after induction of vessel permeability using MCP-1 (5, 10, 50, 100, 250 pg); extracted dye contents were quantified by measuring at 620 nm. Data are expressed as mean ± SEM (n = 10, *P<0.05, t-test).
(2.05 MB TIF)
