# Supplementary figures and images for: Broad Spectrum Antiangiogenic Treatment for Ocular Neovascular Diseases
Source: PLoS One. 2010 Sep 1;5(9):e12515. doi: 10.1371/journal.pone.0012515 (PMC2931703; doi:10.1371/journal.pone.0012515)

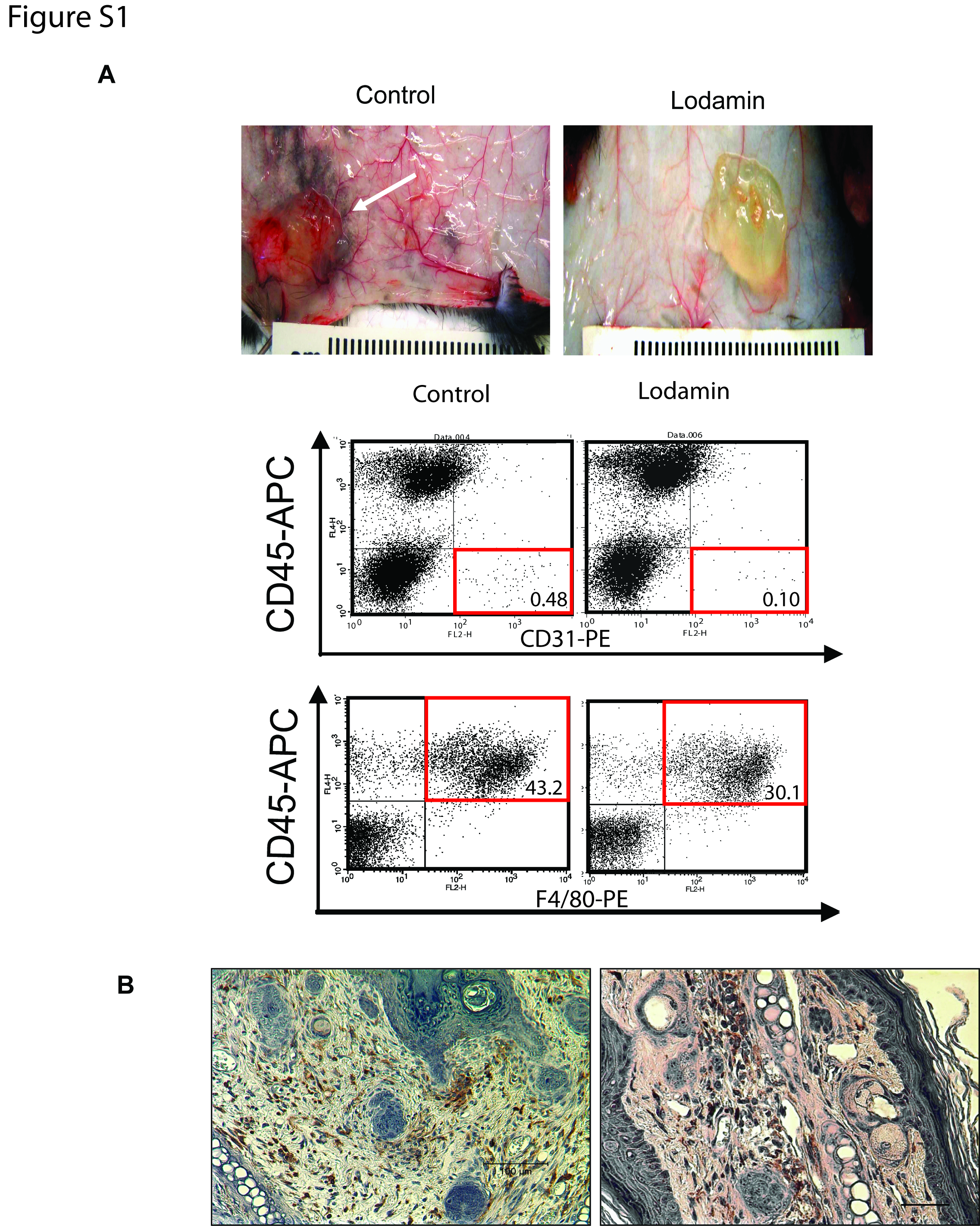

Supplement: Figure S1 — Lodamin inhibits angiogenesis and inflammatory response in Matrigel plug angiogenesis assay and DTH reaction. (A) Upper panel: representative Matrigel plugs exposed under mouse skin (bar = 1 cm). Vehicle treated mice presented bloody plugs surrounded by massive blood vessels, compared to Lodamin treated mice which had poor vasculature. bar = 100µm. Lower panel: FACS dot plots of endothelial cells (CD31+/CD45, marked in red squares) or macrophages (CD45+F4/80+, marked in red squares) from single cell suspension originated from Matrigel plugs. (B) Immunohistological analysis of mice ears post DTH reaction: staining of macrophages using F4/80 marker. Control ears exhibited an excessive inflammation. Bar = 50 µm. No obvious difference was found in macrophage distribution however, the total number of macrophages present in control mice was elevated due to greater amount of tissue, associated with increased swelling. (9.63 MB TIF) [file pone.0012515.s001.tif]

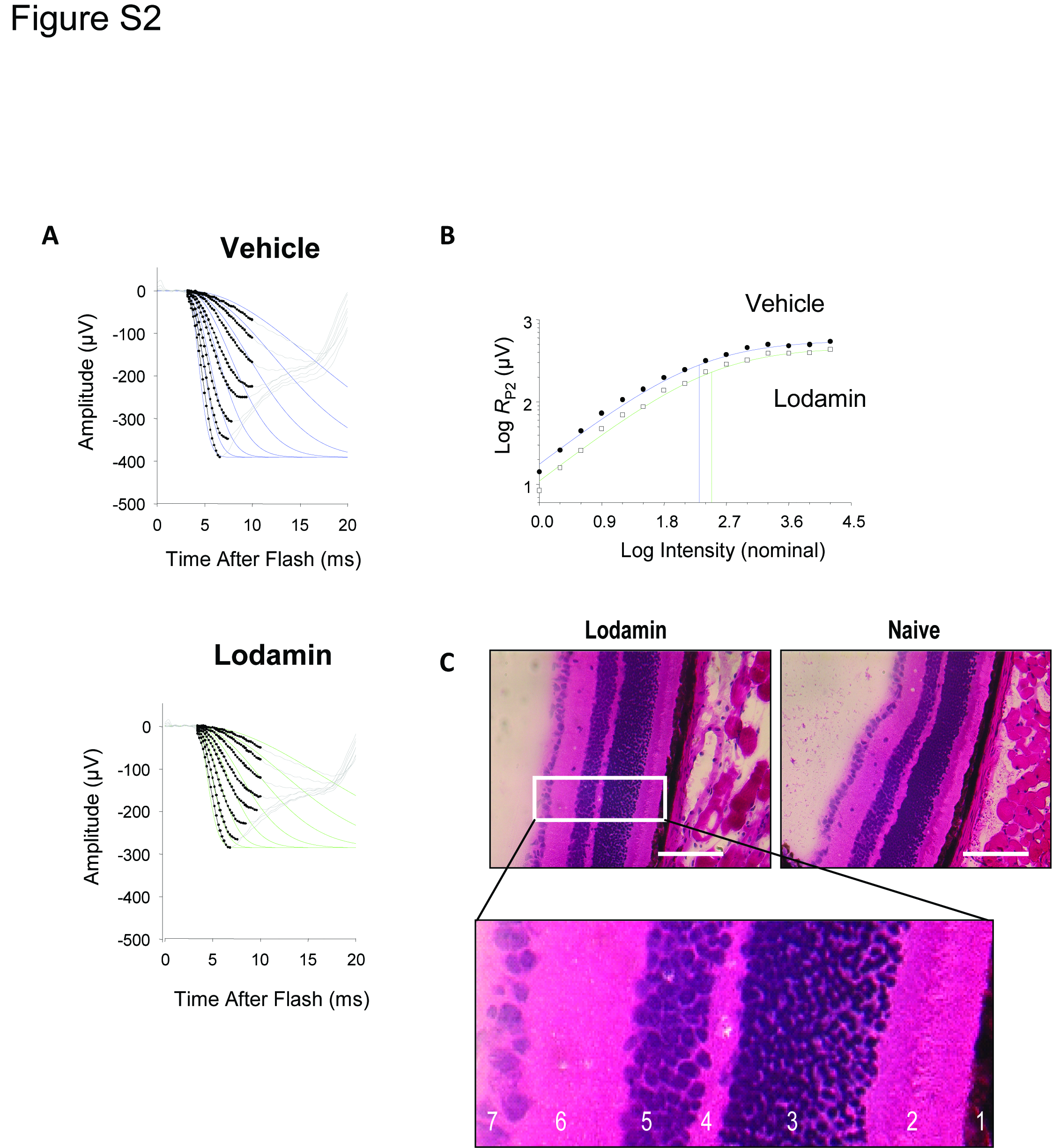

Supplement: Figure S2 — Evaluation of retinal toxicity post intravitreal injection of Lodamin (A)Retinal electroradiography post Lodamin intravitreal injection. The a-waves in response to the eight brightest flashes are replotted at increased gain. The black portions are fitted with the Hood and Birch model of the activation of phototransduction (eq. 1; colored lines). (B) Fits of eq. 2 to the response vs. intensity relationship of P2. Drop lines indicate the intensity that elicits a flash with half-maximum amplitude. (C) Histological cross sections of retinal tissues. Retinal tissues taken 14 days post intravitreal injection of Lodamin (300 µg/eye) were compared to naïve mouse retinas. Representative images are shown (Bars = 50 µm). No apparent retinal tissue changes were detected, both eyes presented normal structures of (1) choroid (2) photoreceptors (3) outer nuclear layer (4)outer plexiform layer (5) inner nuclear layer (6) inner plexiform layer and (7) ganglion cell layer. The total retinal thickness remained unchanged. (5.11 MB TIF) [file pone.0012515.s002.tif]

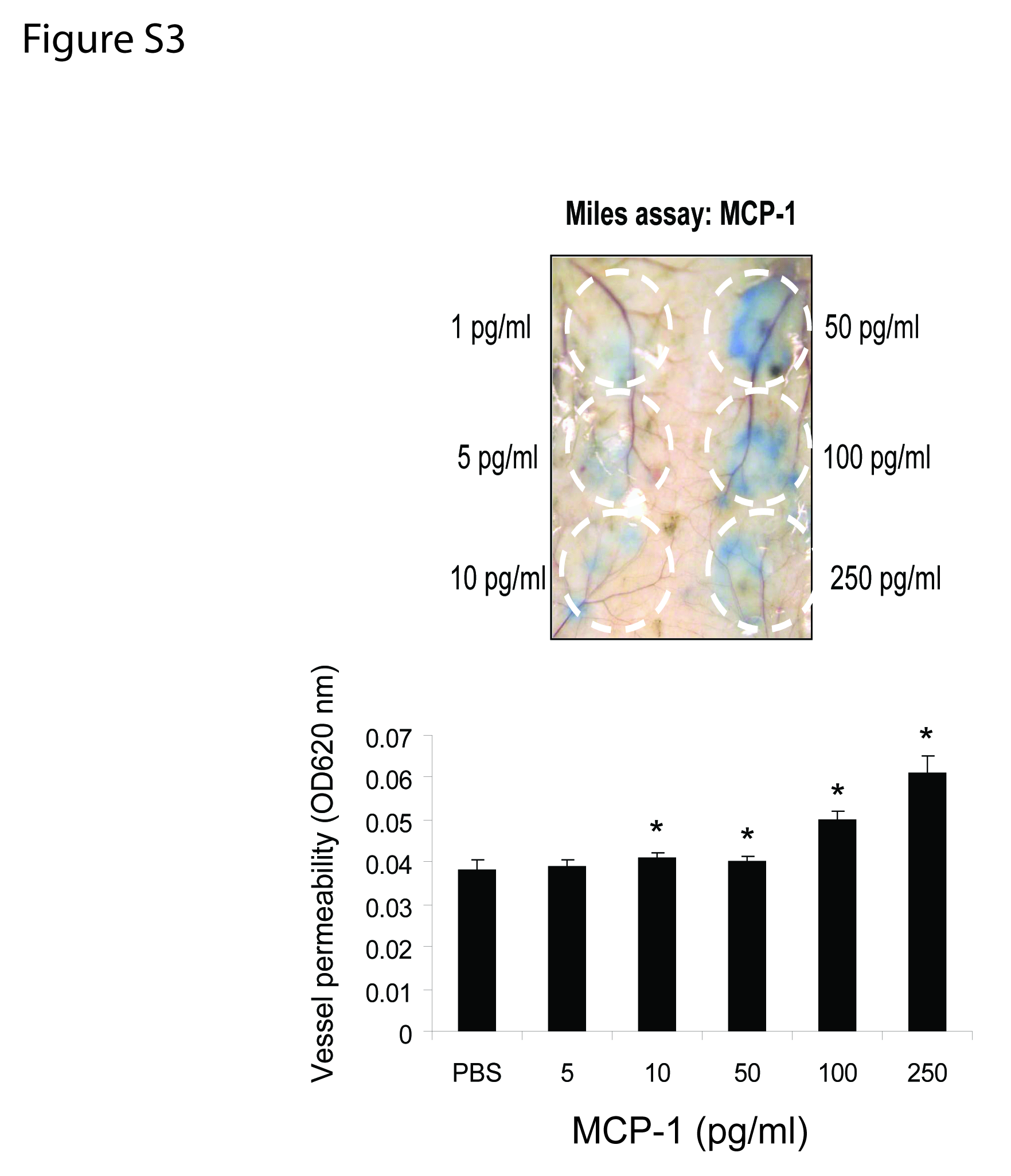

Supplement: Figure S3 — Induction of vessel permeability by MCP-1 demonstrated in Miles assay. Representative photos of mouse skin showing dose response MCP-1-induced vessel permeability. Graph represent the quantification of Miles assays performed in mice after induction of vessel permeability using MCP-1 (5, 10, 50, 100, 250 pg); extracted dye contents were quantified by measuring at 620 nm. Data are expressed as mean ± SEM (n = 10, *P<0.05, t-test). (2.05 MB TIF) [file pone.0012515.s003.tif]
